# Effectiveness of Physical Activity Programs for Older Adults during COVID-19 across Districts with Different Healthcare Resource: A Case Study of Keelung City in Taiwan

**DOI:** 10.3390/healthcare12121177

**Published:** 2024-06-11

**Authors:** Lain-Li Lin

**Affiliations:** Department of Tourism, Leisure and Health, Deh Yu College of Nursing and Health, Keelung City 203, Taiwan; 80202001E@ntnu.edu.tw

**Keywords:** older adults, physical activity, medical resources

## Abstract

While past research has largely supported the importance of physical activity for the health of older adults, during the COVID-19 pandemic, older individuals may have reduced their opportunities and levels of physical activity to lower the risk of infection by minimizing outings or interactions with others. Additionally, the adequacy of medical resources in a region is often closely related to factors such as infrastructure and economic resources. Therefore, it is important to consider whether there are differences in health promotion among older adults living in areas with varying levels of medical resources. This study aimed to implement a physical activity program for older adults, selecting Keelung City, Taiwan, as the implementation area, and comparing the differences in implementation effectiveness among administrative districts with different levels of medical resources in the city. The study employed a two-way analysis of variance to examine the differences in the effectiveness of the physical activity program among administrative districts, where the average service population in medical institutions was higher or lower than the city average. The results revealed significant improvements in participants’ right-hand grip strength and the number of sit-to-stand repetitions in the overall sample after the program intervention. Moreover, in administrative districts where the average service population in medical institutions was higher than the city average, participants showed greater improvement in grip strength, while in districts where it was lower than the city average, participants demonstrated greater improvement in sit-to-stand repetitions. Future research could draw upon these findings to design physical activity programs tailored to different regions with distinct allocations of medical resources. Tailored program designs considering local medical resources are necessary to optimize effectiveness. Governments and organizations should focus on elderly health, especially in resource-scarce areas, by increasing investment and support for these programs to promote well-being and bridge disparities.

## 1. Introduction

With the ongoing improvement in global living environments and advancements in medical standards, people’s quality of life continues to improve and life expectancy is extending [1,2]. Population aging poses challenges to both developing and developed countries, impacting various aspects, such as health care, economics, education, and social welfare [3,4,5]. As individuals age and the aging process progresses, their physiological health gradually declines, including a reduction in muscle mass. Aging is accompanied by decreased physical function and muscle endurance, reduced flexibility, and bone loss, leading to an increased proportion of chronic diseases, affecting walking balance, increasing the risk of falls, and, consequently, impacting quality of life. It is evident that delaying the aging process and maintaining physical function in older adults are important issues. Lack of physical activity or a sedentary lifestyle are some of the causes of global mortality and disease [6,7], including dementia [8,9]. In addition to dementia, there are various other risks, such as stroke [10], heart disease and hypertension [11], and depression [12], highlighting the importance of physical activity for older adults.

With the occurrence of the COVID-19 pandemic, the health issues of older adults have gained increased attention. How to maintain a healthy body to resist diseases and minimize harm has become a question that people constantly ponder. Many studies have pointed out that addressing the decline in physical function among older adults, in addition to medical intervention, incorporating appropriate exercise not only improves deteriorating physiological functions and slows down the aging process, but also helps prevent the risk of cardiovascular and cancer-related diseases [13]. However, many health promotion programs for older adults have been hindered by COVID-19. During the pandemic, governments may have recommended reducing contact among individuals to prevent the spread of the disease. Most health promotion programs are typically conducted in schools, community centers, or relevant institutions. Unfortunately, as with some schools transitioning to online learning, many health promotion activities have been forced to pause or switch to online platforms. However, the digital technology literacy of older adults differs from that of younger student populations, and some older adults may find it challenging to participate in online health promotion activities. Furthermore, as older adults are at a higher risk of severe illness from the pandemic, their families or local governments may discourage them from going out, further hindering their opportunities to participate in health promotion activities. Researchers have observed this phenomenon and attempted to explore the effectiveness of implementing physical activity programs for older adults during the pandemic.

Aoyagi and Shephard [14] found that in societies with an aging population, as individuals aged, their skeletal muscle control and limb coordination declined. This decline leads to older adults being unable to engage in prolonged activities, have difficulty changing direction quickly, cannot stand for long periods, experience reduced balance, and suffer from impaired hand–foot coordination, making them more prone to falls and injuries [15]. The decline in physical activity ability also hinders the completion of daily tasks, affecting the independence and balance ability of older adults, which can lead to accidental falls and fractures [16]. Hurley and Roth [17] pointed out that resistance training can enhance muscle strength, power, and walking speed in older adults, which is one of the important factors in fall prevention. From the perspective of physical fitness, factors related to falls in older adults include muscle strength, power, muscle endurance, and balance, which decline with age, and although aging is inevitable, particularly in terms of dynamic agility, it is difficult to maintain an optimal status even after exercise intervention. Various functions of the body parts of older adults change significantly with age, including a decreased lung capacity, reduced muscle mass, osteoporosis, and kyphosis [18]. The decline in muscle strength and balance function is a major factor leading to an increased risk of chronic diseases and loss of daily self-care ability [18]. Related research also indicates that insufficient balance ability and lower limb strength are among the main factors leading to falls in older adults [19]. Aging also leads to a decrease in the number of motor neurons, muscle atrophy, and limited flexibility, which can result in joint damage and an inability to control body balance, increasing the risk of falls [20].

Exercise programs have a significant impact on the muscle strength and balance ability of older adults, thereby enhancing their functional physical fitness and reducing the risk of falls [21]. When designing exercise programs, maintaining or enhancing muscle strength should be the primary consideration, and they should be carried out according to the principle of progression. The focus of the program should be on training large muscle groups to improve muscle strength, cardiovascular endurance, muscle endurance, and joint flexibility. Nelson et al. [22] suggested that after performing stretching warm-up exercises, strength training and aerobic exercise should be conducted, followed by soothing movements to conclude. Functional physical fitness has also been proven to be an important predictor of the quality of life of older adults [23]. Furthermore, multiple studies have shown that the functional physical fitness of older adults is closely related to the risk of falls, with better physical function being associated with a lower risk of falls [24,25]. After integrating exercise training programs, several physiological indicators of older adults have shown positive improvements. In summary, effective exercise programs can significantly improve the muscle strength, cardiovascular fitness, and balance ability of older adults, thereby improving their functional physical fitness [26].

Government policies, population structure, economic conditions, and geographical location are among the various factors that can lead to differences in the allocation of medical resources among different administrative regions. This imbalance may result in some areas lacking sufficient medical facilities, healthcare personnel, and medical technology, thereby limiting the access of older adults to different levels of medical services. Particularly during epidemics or other major events, these essential resources may become even scarcer [27]. In areas with limited medical resources, due to shortages of hospitals, nursing homes, and medical centers, older adults may not receive timely health checks, treatments, and related services. This could lead to health problems not being detected and addressed in a timely manner, thereby affecting health outcomes. It can be seen that the allocation of medical resources is closely related to the health issues of older adults. Furthermore, areas with limited medical resources may also face economic difficulties and an inadequate infrastructure [28], further restricting the development and participation of elderly health promotion activities. For example, a lack of exercise facilities and fitness equipment may hinder the willingness and ability of older adults to participate in sports and physical activities [29], thereby potentially affecting their health. Therefore, this study aims to investigate whether the implementation of physical activity programs in areas with different medical resources would lead to differences in effectiveness. Hence, the objectives of this study are as follows:

1. Implement a physical activity and investigate its effectiveness in improving the physical function of older adults during the pandemic.

2. Compare whether there are different outcomes in different regions after implementing physical activity in areas with different medical resources during the pandemic.

## 2. Materials and Methods

### 2.1. Participants

This study included a total of 166 participants, aged between 58 and 88 years (mean age = 72.87; *SD* = 6.69 years; 21% were male), from different administrative districts within Keelung City, Taiwan. Researchers recruited older adults from various administrative districts in Keelung City willing to participate in the physical activity promotion program. Recruitment was conducted through posters placed on community bulletin boards, and interested participants could sign up by contacting the researchers using the information provided on the posters. Ultimately, participants were recruited from one to two communities in each administrative district. Table 1 shows the sources, ages, and distribution of the participants. For research ethics considerations, the current study assigned de-identified codes to each administrative district and community. Additionally, Table 1 displays the average number of medical institution services per administrative district (administrative district population divided by the number of medical institutions), coded based on government announcements (approximate figures provided for de-identification purposes only). Administrative districts with service numbers above the Keelung City average were coded as 1, while those below the Keelung City average were coded as 0, based on the research objectives.

### 2.2. Design and Procedures

This study adopted a quasi-experimental design and used stratified convenience sampling to recruit participants from different administrative districts. Due to practical considerations, the threshold for participating in this study was relatively high, as participants had to fully engage in the study and complete both pre-tests and post-tests. Therefore, this study lacked the requirement for random assignment, having only experimental and control groups with pre-test and post-test measurements. Firstly, participants recruited by researchers attended a pre-study meeting conducted by the researchers themselves to explain the study procedures and data collection protocols. Upon confirming their willingness to participate, participants were provided with an informed consent form to sign. After completing this process, researchers measured participants’ muscle strength and performance-related indicators, including grip strength, the number of chair stands in 30 s, and the time taken to walk 6 m. The formal study then commenced after the pre-study procedures were completed.

The formal study consisted of a 12-week physical activity program, with two hours of training per week, totaling 24 h. The program was led by a head coach with a master’s degree in sports science and experience in leading physical activities for older adults. Based on the predictive data measured by the researchers, the coach assessed the participants’ physical health and fitness levels and designed appropriate muscle strength and endurance training programs. The physical activity program implemented in this study was primarily based on the design by Lin and Liu [30]. The program included upper and lower limb muscle strength and endurance training, core muscle group exercises, and was designed according to the guidelines of the American College of Sports Medicine, including exercise mode, progression rate, frequency, intensity, and duration. Since older adult participants in this study did not have a habit of regularly engaging in resistance or weight training, high-intensity resistance (>70% 1RM) and weight training were not performed. Instead, the focus was on improving muscle endurance (30–60% 1RM) or resistance and weight training for body weight and core exercises. The program included both dynamic and static stretching to increase joint mobility and aimed to increase participants’ muscle strength and endurance to adapt to and meet their daily needs (such as dressing, lifting objects, and fall prevention). The program content was arranged with items suitable for older adults, emphasizing lightness, accessibility, and minimal risk of injury (such as water bottles for weight, resistance bands, yoga balls, etc.). Finally, post-tests were conducted after the program ended to assess the effectiveness of the exercise plan.

### 2.3. Measurement of Variables

This study included three measurement variables (namely, handgrip strength, the number of chair stands in 30 s, and the time required for a 6 m walk), as well as data from the Taiwan Ministry of Health and Welfare for the year 2021 (i.e., the average service population for each medical institution). Firstly, the purpose of the handgrip test was to measure the maximum grip strength of both hands, which is an indicator of muscle strength for assessing muscle atrophy. During the test, researchers adjusted the grip of the handgrip dynamometer so that the participant’s second finger joint formed a right angle. Then, participants naturally extended their arms, looked straight ahead, and gripped forcefully. Additionally, the handgrip dynamometer could not touch the body, and the results were measured in kilograms.

Secondly, the number of chair stands in 30 s was recorded by the researchers, with participants attempting to stand up and sit down as many times as possible within 30 s. A complete stand-up and sit-down cycle was counted as one repetition. Lastly, the purpose of measuring the time taken for a 6 m walk was to assess the difficulty of walking in daily life, which is an important indicator of physical performance for evaluating muscle atrophy. Researchers first drew four parallel lines on the ground (start, 1 m, 7 m, and 8 m), with participants standing at the starting point. Upon the researcher’s instruction, participants needed to walk from the starting point to the mark at 8 m, and the researcher recorded the time it takes for participants to walk from the 1 m mark to the 7 m mark. Finally, the data source for the average service population for each medical institution was the Taiwan Ministry of Health and Welfare. Researchers coded administrative districts with populations below the city average as 0 and those above the city average as 1 for the subsequent data analysis.

### 2.4. Data Analysis

The current study included descriptive statistics and a two-factor mixed-design analysis of variance (ANOVA). Descriptive statistics were used to present the pre/post-test score before/after the muscle training program for participants in the lower/higher average number of service population per medical institution groups separately. The two-factor mixed-design ANOVA was used to analyze whether there were differences in the pre- and post-test differences between the two groups of participants in grip strength, 30 s sit-to-stand, and 6 m walking time.

## 3. Results

### 3.1. Descriptive Statistics

The results of the descriptive statistics are presented in Table 2. Firstly, the average service population of each medical institution was divided into two groups: 67 participants in the group with service population averages lower than the Keelung City average and 99 participants in the group with service population averages higher than the Keelung City average. Compared to the group with lower service population averages, the group with higher service population averages may have had fewer medical resources allocated per person, which formed the basis of grouping in this study. Additionally, the mean and standard deviations of each measurement variable were listed based on the group and pre/post-test, facilitating the interpretation of the subsequent research results.

### 3.2. Two-Factor Mixed-Design ANOVA

The current study used a two-factor mixed-design ANOVA to analyze the promotion effect of the muscle-strength training program on the physical activity performance of participants in terms of the grip strength, 30 s sit-to-stand test, and 6 m walking time, before and after the intervention, as well as the average number of people served by each medical institution. The average number of people served by each medical institution was an independent sample variable, and the comparison between the pre-test and post-test was a dependent sample variable. The analysis results are shown in Table 3. First, in the right-hand grip strength, all participants showed a significant improvement after the intervention, *F*(1, 164) = 22.38, *p* < 0.000; in the left-hand grip strength, all participants showed no significant improvement after the intervention, *F*(1, 164) = 1.19, *p* = 0.277; in the 30 s sit-to-stand test, all participants showed a significant improvement after the intervention, *F*(1, 164) = 82.88, *p* < 0.000; in the 6 m walking time, all participants showed no significant improvement after the intervention, *F*(1, 164) = 0.58, *p* = 0.446.

**Figure 1:**
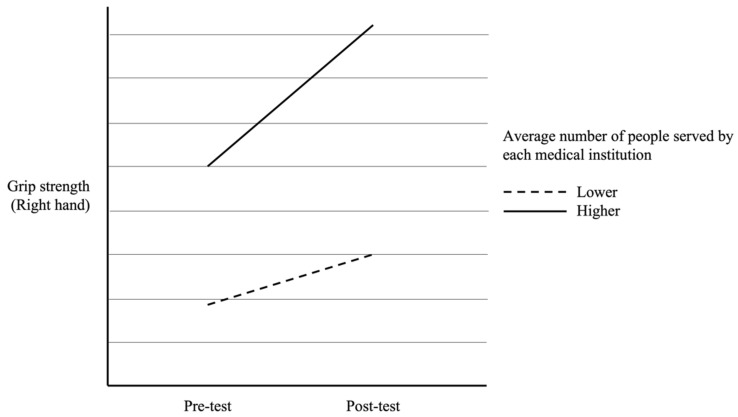
The difference in pre- and post-test right-hand grip strength between participants with different average numbers of people served by each medical institution.

**Figure 2:**
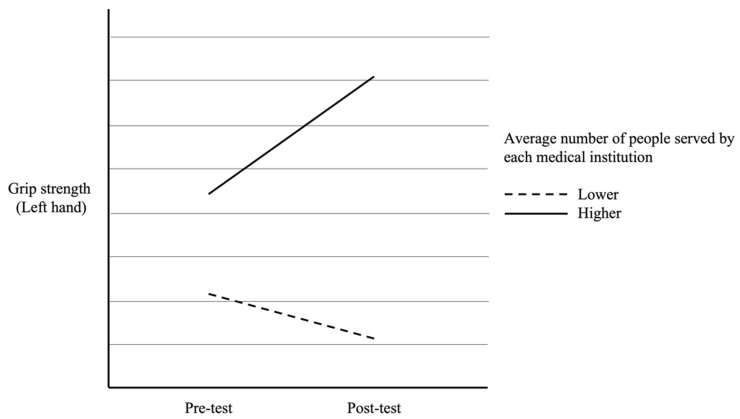
The difference in pre- and post-test left-hand grip strength between participants with different average numbers of people served by each medical institution.

**Figure 3:**
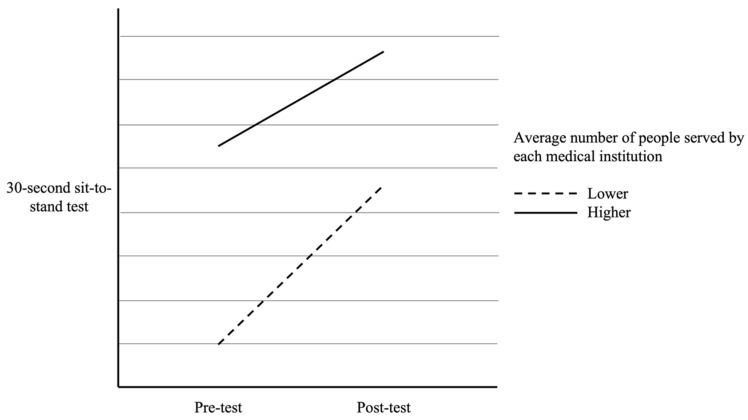
The difference in pre- and post-test 30 s sit-to-stand test between participants with different average numbers of people served by each medical institution.

In addition, as seen in Table 3, there were significant interaction effects for participants from different average number of people served by each medical institution on pre- and post-test scores of right-hand grip strength [*F*(1, 164) = 4.75, *p* = 0.031], left-hand grip strength [*F*(1, 164) = 6.11, *p* = 0.014], and 30 s sit-to-stand test [*F*(1, 164) = 5.43, *p* = 0.021]. The interaction effects were visually presented in Figure 1, Figure 2 and Figure 3. However, the interaction effect was not significant in the 6 m walking test [*F*(1, 164) = 3.18, *p* = 0.077]. Specifically, participants from medical institutions with a higher average number of service population showed a greater improvement in grip strength after the muscle strength training program intervention (as shown in Figure 1 and Figure 2). Although all participants showed a significant improvement in the 30 s sit-to-stand test, those from medical institutions with a lower average number of service population demonstrated a greater improvement after the intervention (as shown in Figure 3).

## 4. Discussion

Implementing health-promotion-related programs is one of the significant pathways to enhance the health of older adults, particularly highlighted during the COVID-19 pandemic. This study aimed to investigate the impact of implementing physical activity programs on the functional fitness of older adults during the pandemic. The research findings indicated that implementing such programs during the pandemic significantly improved the muscle strength and physical functioning of older adults. Specifically, all participants exhibited a significant improvement in right-hand grip strength after the intervention. Additionally, participants from administrative districts with a higher average service population from medical institutions showed more significant improvements in muscle strength, suggesting a possible association between the disparities in medical resource allocation during the pandemic and training effectiveness. Therefore, the following discussion detailed the findings of this study.

The current research findings revealed significant improvements in participants’ right-hand grip strength and sit-to-stand test scores after the intervention, consistent with some previous studies. For instance, past research showed that physical activity programs could significantly improve muscle strength [31] and balance [32] in older adults. However, interestingly, studies on implementing physical activity programs for older adults suggested that shorter-term interventions may not necessarily lead to significant improvements in functional fitness. For example, in the study by Ordnung et al. [33], a 6-week exercise game training only resulted in significant improvements in fine motor skills in older adults’ left hands, while other indicators (such as flexibility, balance, or grip strength) did not show statistically significant progress. Therefore, based on the non-significant improvements observed in certain aspects among participants after the intervention in this study, it is suggested that the effects of relatively long-term physical activity programs may be more comprehensive, and the duration of the program implemented in this study might not have been sufficient to demonstrate differences in these aspects. Particularly for older adults, maintaining long-term exercise habits is crucial for improving and maintaining their physical function and overall quality of life. Hence, future research may consider implementing longer intervention programs to further improve the physical function and health of older adults.

Furthermore, the research findings also indicated that participants in administrative districts with a higher average service population (meaning a larger population served per medical institution) showed significantly greater improvements in grip strength of both hands after the intervention. Based on previous research findings, it could be inferred that medical service institutions in areas with scarce resources may face certain problems, such as shortages in human resources, inadequate infrastructure, policy issues, and transportation inconvenience [34,35,36], and these areas may also have lower levels of urbanization. Areas with lower levels of urbanization often experience problems such as an outflow of the young population and an increase in the elderly population, which may urgently require measures related to elderly support [37]. Therefore, the results of this study also suggested that implementing physical activity programs in areas with relatively fewer medical resources was effective, with even more prominent improvements were observed in upper limb strength. Such results could be explained by the psychology of the scarcity principle, where people tend to value and cherish resources more when they become scarce [38]. In areas with fewer resources, people may face more restrictions and competition, hence, devoting greater efforts to utilizing and protecting existing resources to meet their needs. This may lead to differences in learning outcomes of the same physical activity program entering communities in different areas.

## 5. Limitations and Future Directions

Regarding the limitations and recommendations of the study, it was constrained by time and resource limitations, thus, it was unable to recruit participants from a nationwide scope and compare their differences. Future researchers are encouraged to explore relevant issues in different regions to compare regional disparities. Additionally, in terms of data collection, this study only measured a portion of basic physiological functions. Therefore, future research should consider measuring other physiological or psychological variables to further expand the content and findings of related studies. In terms of research methods, this study did not achieve random sampling, and the sample only represented older adults in the specific administrative district. Future research should target different regions and areas with varying healthcare resource distributions to provide a more comprehensive explanation of the related issues. Future researchers are also encouraged to use longitudinal data collection methods and include more diverse samples to gather more information on the changes in basic physical functions of older adults. Finally, through the research findings, we also recommend that government agencies or other relevant practitioners provide relevant health promotion programs in areas where medical resources are relatively scarce.

## 6. Conclusions

This study found that implementing physical activity programs for older adults in areas with different levels of medical resources yielded varying degrees of effectiveness. Regarding the first research objective, the results demonstrated that physical activity programs could effectively improve the physical function of older adults during the pandemic. The improvements were observed in key physical metrics such as the grip strength (left hand) and 30 s sit-to-stand test. For the second research objective, the comparison between different regions revealed that the effectiveness of these programs varied depending on the availability of medical resources. This disparity highlighted the necessity for tailored program designs that consider local medical resource availability to optimize effectiveness.

Furthermore, the researchers emphasize the importance of providing appropriate physical activity programs for older adults, especially those with limitations in daily functioning, in regions with relatively scarce resources. The design of these programs should account for the local medical resource situation and be adjusted and optimized according to local needs to ensure both effectiveness and sustainability. Meanwhile, for areas with relatively scarce medical resources, governments and relevant organizations should strengthen their focus on elderly health, increase investment, and support corresponding physical activity programs. Such initiatives can significantly promote the health and well-being of older adults in these regions, bridging the gap caused by disparities in medical resource distribution.

## Figures and Tables

**Table 1:** Age distribution of participants, distribution by administrative region, and average number of patients served by medical institutions in each administrative region.

Administrative District (Code)	Community (Code)	N	Age Range (*M*/*SD*)	Average Number of People Served by Medical Institutions (Coding)
A	a	19	61~88 (75.42/8.05)	Approximately 3000 people (1)
	b	20	65~87 (72.70/6.81)	
B	a	31	58~87 (71.59/7.79)	Approximately 2500 people (1)
	b	20	65~88 (72.75/6.92)	
C	a	21	65~81 (71.00/5.47)	Approximately 1500 people (0)
	b	16	65~87 (75.63/6.50)	
D	a	12	66~83 (75.50/4.96)	Approximately 750 people (0)
E	a	18	66~80 (72.33/4.50)	Approximately 500 people (0)
F	a	9	65~75 (69.22/3.38)	Approximately 2500 people (1)

**Table 2:** Results of descriptive statistics.

Average Number of People Served by Each Medical Institution	n	Grip Strength (Right Hand)		Grip Strength (Left Hand)		30 s Sit-to-Stand Test		6 m Walking	
		Pre-Test (kg)	Post-Test (kg)	Pre-Test (kg)	Post-Test (kg)	Pre-Test (Times)	Post-Test (Times)	Pre-Test (s)	Post-Test (s)
		*M* (*SD*)	*M* (*SD*)	*M* (*SD*)	*M* (*SD*)	*M* (SD)	*M* (*SD*)	*M* (*SD*)	*M* (*SD*)
Lower average number of service population per medical institution	67	19.89 (6.78)	20.64 (6.87)	19.63 (7.00)	19.23 (5.85)	16.46 (5.17)	19.70 (5.87)	5.51 (1.83)	5.79 (2.21)
Higher average number of service population per medical institution	99	21.92 (7.19)	23.95 (6.55)	20.51 (7.32)	21.54 (6.62)	20.52 (6.24)	22.43 (6.02)	4.70 (1.25)	4.59 (1.25)

**Table 3:** Results of two-factor mixed-design ANOVA.

	Source of Variation	*SS*	*df*	*MS*	*F*	*p*
Grip strength (right hand)	Between groups	571.17	1	571.17	6.56	0.011
	Error	14,288.70	164	87.13		
	Within groups	153.52	1	153.52	22.38	<0.001
	Between × Within	32.55	1	32.55	4.75	0.031
	Error	1124.84	164	6.86		
Grip strength (left hand)	Between groups	203.80	1	203.80	2.40	0.124
	Error	13,947.06	164	85.04		
	Within groups	7.99	1	7.99	1.19	0.277
	Between × Within	40.99	1	40.99	6.11	0.014
	Error	1100.22	164	6.71		
30 s sit-to-stand test	Between groups	919.84	1	919.84	14.58	<0.001
	Error	10,345.97	164	63.09		
	Within groups	531.54	1	531.54	82.88	<0.001
	Between × Within	34.79	1	34.79	5.43	0.021
	Error	1051.77	164	6.41		
6 m walking	Between groups	81.04	1	81.04	19.20	<0.000
	Error	692.11	164	4.22		
	Within groups	0.57	1	0.57	0.58	0.446
	Between × Within	3.09	1	3.09	3.18	0.077
	Error	159.67	164	0.97		

Note: The symbol “×” indicates the interaction effect between the between-group and within-group variables.

## Data Availability

The raw data supporting the conclusions of this article will be made available by the author without undue reservation. The data are not publicly available due to restrictions on their containing information that could compromise the privacy of the research participants.
